# Plant-Type Trehalose Synthetic Pathway in *Cryptosporidium* and Some Other Apicomplexans

**DOI:** 10.1371/journal.pone.0012593

**Published:** 2010-09-07

**Authors:** Yonglan Yu, Haili Zhang, Guan Zhu

**Affiliations:** 1 College of Veterinary Medicine, China Agricultural University, Beijing, China; 2 Department of Veterinary Pathobiology, College of Veterinary Medicine & Biomedical Sciences, Texas A&M University, College Station, Texas, United States of America; 3 Faculty of Genetics Program, Texas A&M University, College Station, Texas, United States of America; INSERM U1016, Institut Cochin, France

## Abstract

**Background:**

The trehalose synthetic pathway is present in bacteria, fungi, plants and invertebrate animals, but is absent in vertebrates. This disaccharide mainly functions as a stress protectant against desiccation, heat, cold and oxidation. Genes involved in trehalose synthesis have been observed in apicomplexan parasites, but little was known about these enzymes. Study on trehalose synthesis in apicomplexans would not only shed new light into the evolution of this pathway, but also provide data for exploring this pathway as novel drug target.

**Methodology/Principal Findings:**

We have observed the presence of the trehalose synthetic pathway in *Cryptosporidium* and other apicomplexans and alveolates. Two key enzymes (trehalose 6-phosphate synthase [T6PS; EC 2.4.1.15] and trehalose phosphatase [TPase; EC 3.1.3.12] are present as Class II bifunctional proteins (T6PS-TPase) in the majority of apicomplexans with the exception of *Plasmodium* species. The enzyme for synthesizing the precursor (UDP-glucose) is homologous to dual-substrate UDP-galactose/glucose pyrophosphorylases (UGGPases), rather than the “classic” UDP-glucose pyrophosphorylase (UGPase). Phylogenetic recontructions indicate that both T6PS-TPases and UGGPases in apicomplexans and other alveolates are evolutionarily affiliated with stramenopiles and plants. The expression level of *T6PS-TPase* in *C. parvum* is highly elevated in the late intracellular developmental stage prior to or during the production of oocysts, implying that trehalose may be important in oocysts as a protectant against environmental stresses. Finally, trehalose has been detected in *C. parvum* oocysts, thus confirming the trehalose synthetic activity in this parasite.

**Conclusions/Significance:**

A trehalose synthetic pathway is described in the majority of apicomplexan parasites including *Cryptosporidium* and the presence of trehalose was confirmed in the *C. parvum* oocyst. Key enzymes in the pathway (i.e., T6PS-TPase and UGGPase) are plant-type and absent in humans and animals, and may potentially serve as novel drug targets in the apicomplexans.

## Introduction

Trehalose (*α*-D-glucopyranosyl-1,1-*α*-D-glucopyranoside) is a disaccharide consisting of two units of glucose linked by an *α*,*α*-1,1-glycosidic linkage. It is present in a wide range of organisms, including prokaryotes, fungi, plants, and invertebrate animals [1]–[4]. Trehalose may serve as an energy source, but its major function is known as a protectant against various stresses including desiccation/dehydration, heat, cold and oxidation [5], [6]. Additionally, trehalose may be an integral component of cell wall glycolipids in mycobacteria and corynebacteria [7], and the pathway and intermediary metabolites may also play regulatory roles in signaling, sugar metabolism or stress-responses [1]–[5], [8]–[13].

**Figure 1:**
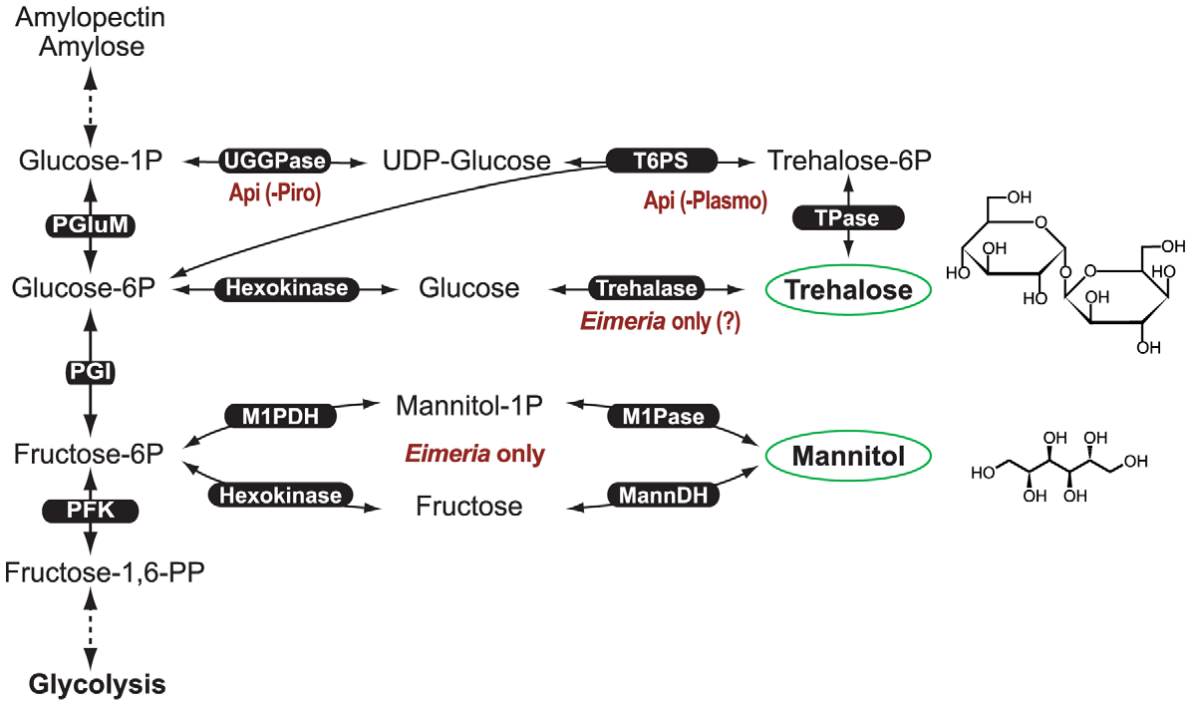
Presence of trehalose synthetic pathway in the apicomplexans as determined from available genome sequences. Within the pathway, UDP-glucose/galatose pyrophosphorylase (UGGPase) is present in almost all apicomplexans with the exception for piroplasmids (e.g., *Theileria* and *Babesia*) [marked as Api(-Piro)], while trehalose-6P synthase and trehalose phosphatase (TPase) are fused as a bifunctional protein that is present in all apicomplexans with the exception for *Plasmodium* species [marked as Api(-Plasmo)]. Trehalase may only be present in the intestinal coccidia (*Eimeria*). For comparison, the mannitol cycle present only in the intestinal coccidia (represented by *Eimeria tenella*) is also illustrated.

Bacteria, fungi, plants and invertebrates can synthesize trehalose from UDP-glucose by trehalose 6-phosphate synthase (**T6PS or TPS**, EC 2.4.1.15) and trehalose phosphatase (**TPase or TP**, EC 3.1.3.12) (**Figure 1**). Vertebrates including humans and other mammals are incapable of synthesizing, but able to metabolize this disaccharide by possessing a trehalase (EC 3.2.1.28). T6PS and TPase can be discrete enzymes or fused together as bifunctional proteins, which, in plants, are referred to as Class I and Class II enzymes, respectively [14], [15].

The Phylum Apicomplexa is comprised of unicellular parasites including many important pathogens in humans and animals, such as *Plasmodium, Babesia, Theileria, Toxoplasma, Eimeria* and *Cryptosporidium* [16]–[18]. This group of protists are evolutionarily close to dinoflagellates and ciliates, and these three phyla form a super group termed alveolates (Alveolata) [18], [19]. The capacity for trehalose synthesis in apicomplexans was first recognized when a gene encoding a T6PS domain was identified in the *Cryptosporidium* genome [20], [21]. Trehalose synthetic genes were also observed in the *Theileria* and *Babesia* genomes [22]–[24]. Trehalose may act as a stress protectant in these parasites, particularly for the oocyst stage in the natural environment (e.g., *Cryptosporidium*) or the sexual developmental stage in vectors (e.g., *Theileria* and *Babesia*). Therefore, it can be reasonably assumed that trehalose may play an important role in protecting apicomplexans from various stresses in their complex life cycle. However, virtually nothing is known on the molecular and biochemical features of this important pathway in apicomplexans, and related genes are also not well annotated in the genome databases.

In the present study we annotated all available apicomplexan genomes and performed phylogenetic reconstructions to delineate the evolutionary history of the key enzymes in the trehalose synthetic pathway. Trehalose has been detected in *C. parvum* oocysts, thus confirming that this pathway is active in this apicomplexan. We show that the synthesis of trehalose in the apicomplexans is mediated by a single bifunctional T6PS-TPase that is evolutionarily affiliated with plant Class II enzymes. Considering that the anti-stress mannitol cycle has been proven to be a drug target in the coccidian *Eimeria* [25], [26], we speculate that the unique, plant-type trehalose synthesis may also be explored as a novel drug target in the apicomplexans.

## Results and Discussion

### Genomic evidence of trehalose synthetic pathway in the majority of apicomplexan lineages except for the *Plasmodium*

Trehalose is synthesized by two reactions, in which T6PS first converts UDP-glucose to trehalose 6-phosphate and this product is in turn converted to trehalose by TPase (**Figure 1**). The production of UDP-glucose from glucose 1-phosphate can be mediated by a classic UDP-glucose pyrophosphorylase (UGPase, EC 2.7.7.9; also termed UTP-glucose-1-phosphate uridylyltransferase) [27], [28], or by a UDP-galactose/glucose pyrophosphorylase (UGGPase, EC 2.7.7.64) that is dual-functional and capable of synthesizing both UDP-glucose and UDP-galactose [29]. Genome analysis indicates the presence of UGGPase among alveolates including the majority of apicomplexan species, with the exception of *Theileria* and *Babesia*; the dinoflagellate *Perkinsus marinus*; and ciliates such as *Paramecium* and *Tetrahymera* (**Table 1**). All aveolates, however, lack the classic UGPase, whereas humans and other mammals possess both UGPase and UAP, but lack UGGPase. Among apicomplexans, the lack of UGPase and UGGPase in *Theileria* and *Babesia* suggests that these two piroplasmids may rely on host cells to supply UDP-glucose, or there might be an unknown pathway to synthesize this nucleotide sugar.

**Table 1:** Evidence of trehalose synthetic pathways in *Cryptosporidium* and some other apicomplexans and alveolates as shown by the presence of genes encoding UDP-galactose/glucose pyrophosphorylase (UGGPase), Class II trehalose-6P synthase-trehalose phosphatase (T6PS-TPase) in their genomes.

		Trehalose metabolism				Mannitol cycle		
Group	Genus	UGPase	UGGPase	T6PS-TPase	Trehalase	M1PDH	M1Pase	MannDH
	(No. species with available genomes)	2.7.7.9	2.7.7.64	2.4.1.15 3.1.3.12	3.2.1.28	1.1.1.17	3.1.3.22	1.1.1.255
Apicomplexan	*Cryptosporidium (3)*	**-**	**+**	**+**	**-**	**-**	**-**	**-**
	*Plasmodium (7)*	**-**	**+**	**-**	**-**	**-**	**-**	**-**
	*Theileria (2)*	**-**	**-**	**+**	**-**	**-**	**-**	**-**
	*Babesia (1)*	**-**	**-**	**+**	**-**	**-**	**-**	**-**
	*Toxoplasma (1)*	**-**	**+**	**+**	**-**	**-**	**-**	**-**
	*Eimeria (1)*	**-**	**+**	**+**	**+**	**+**	**+**	**+**
Dinoflagellate	*Perkinsus (1)*	**-**	**+**	**+**	**+**	**-**	**-**	**-**
Ciliate	*Paramecium (1)*	**-**	**+**	**+**	**+**	**-**	**-**	**-**
	*Tetrahymera (2)*	**-**	**+**	**+**	**-**	**-**	**-**	**-**
Mammal	*Homo (1)*	**+**	**-**	**-**	**+**	**-**	**-**	**-**

**Notes:** Genes involved in the anti-stress related mannitol cycle were also minded for comparison. Plus and minus symbols indicate the presence and absence of specified genes in various apicomplexans, a dinoflagellate and two ciliates at genus level. Humans are listed to represent mammalian hosts for apicomplexan parasites.

UGGPase belongs to a glycosyltransferase family A (GTA) group that also includes UDP-*N*-acetylglucosamine pyrophosphorylase (**UAP**) and other glycosyltransferases [29]. This grouping might explain why some UGGPase genes, such as those from *C. parvum* (GenBank No. XP_628360; locus_tag, cdg7_1830), *T. gondii* (e.g., XP_002370608 and EEB03468), *Perkinsus* (EER18291) and some other eukaryotes are annotated as **UAP** family proteins. Although UAP (EC 2.7.7.23) is evolutionarily related to UGGPases (see phylogenetic data below for more detail), it catalyzes the formation of UDP-*N*-acetyl-α-*D*-glucosamine from *N*-acetyl-α-*D*-glucosamine 1-phosphate and UTP [30]–[33]. On the other hand, *Cryptosporidium* and many other alveolates indeed possess authentic UAP orthologs in their genomes (e.g., XP_625683 in *C. parvum*, XP_665528 in *C. hominis*, and XP_002140971 in *C. muris*).

**Figure 2:**
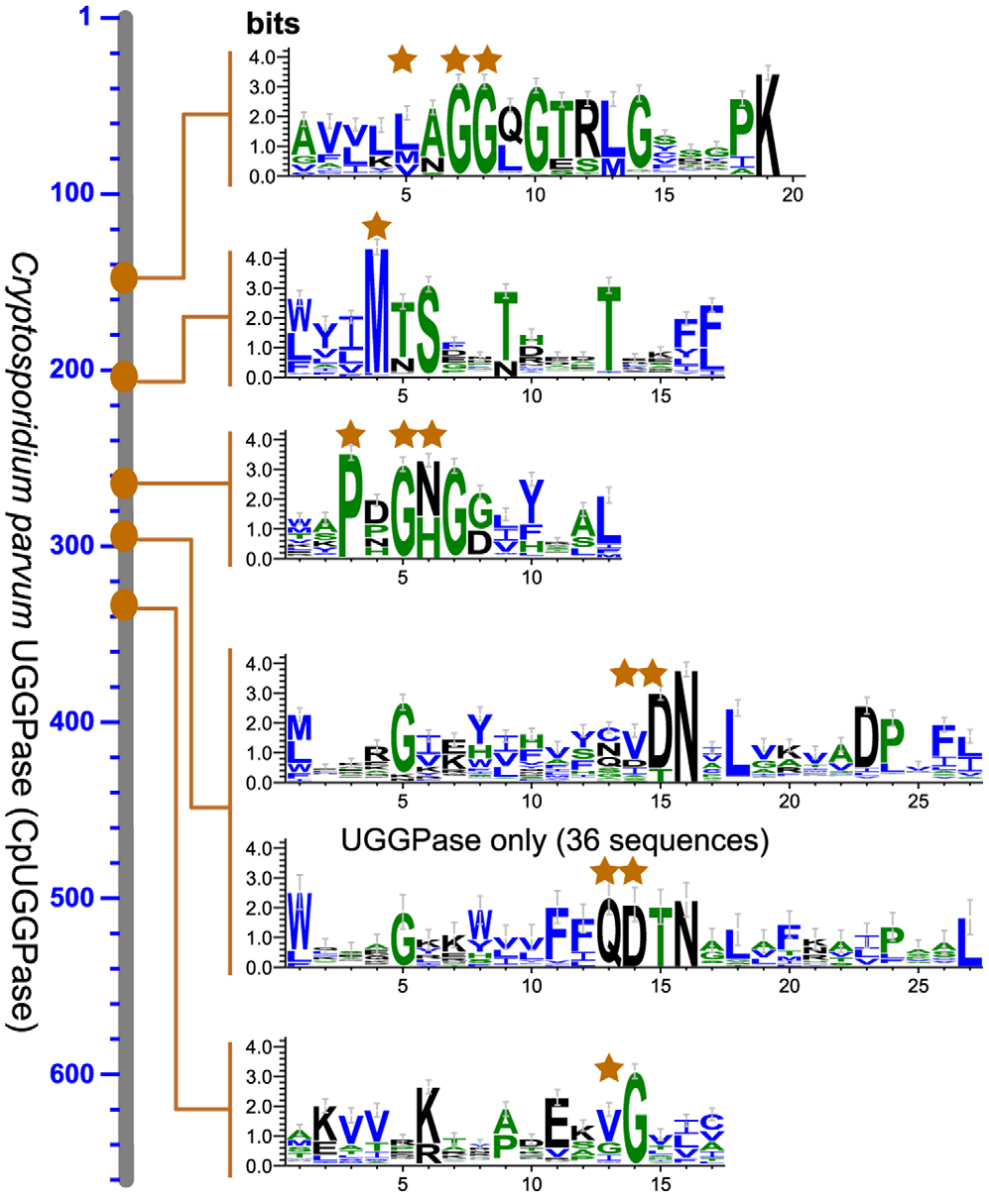
Structure of apicomplexan UDP-glucose/galatose pyrophosphorylase (UGGPase) as exemplified by *Cryptosporidium parvum* protein (CpUGGPase). Sequence logos represent conserved motifs and domains as determined from 224 sequences of glucosyltransferase family-A (GTA) proteins including UGGPases and UDP-*N*-acetylglucosamine pyrophosphorylases (UAPs). In the fourth domain, UGGPases displayed a very unique sequence pattern that differs significantly from other GTA proteins. Stars indicate amino acids important at the active sites.

UGGPases in *Cryptosporidium* and other apicomplexans consist of more than 650 amino acids (aa), and contain all putative active site motifs conserved among the GTA proteins (**Figure 2**). Among the 5 motifs, the fourth one in the apicomplexans is highly distinct from other GTA proteins in their amino acid compositions, from which we predict that this motif is probably important in defining the substrate preferences of this superfamily of enzymes.

**Figure 3:**
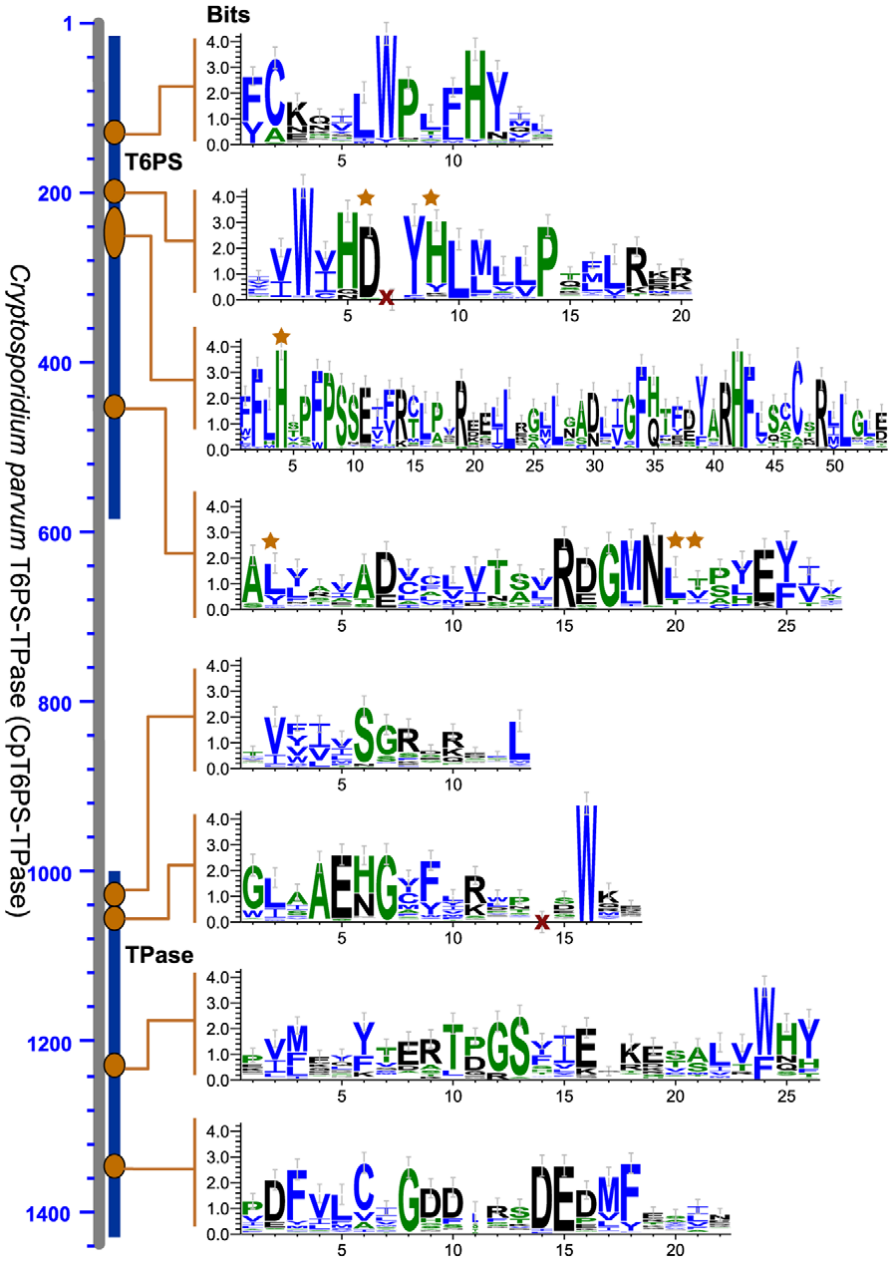
Structure of putative apicomplexan Class II, bifunctional trehalose-6P synthase–trehalose phosphatase (T6PS-TPase) as exemplified by *Cryptosporidium parvum* protein (CpT6PS-TPase). Sequence logos corresponding to the T6PS and TPase domains represent conserved motifs determined from 140 orthologs. Stars indicate amino acids important at the active sites.

Two enzymes (i.e., T6PS and TPase) are involved in converting UDG-glucose to trehalose (**Figure 1**), in which T6PS is a member of the glucosyltransferase family-B (GTB) proteins [34], [35], while TPase belongs to HAD superfamily type IIB enzymes [36]. Nearly all alveolates for which genome data are available possess a putative bifunctional protein containing both T6PS and TPase domains (**Table 1** **and** **Figure 3**). The only exception is *Plasmodium* that lacks either bifunctional or discrete trehalose synthetic enzymes, although their genomes encode UGGPases. Due to the limitation of genome data, it is yet unclear whether other haemosporida also lack trehalose synthesis. Genes encoding T6PS-TPases are intronless in *Cryptosporidium*, but contain introns in other apicomplexans. Conceptually translated apicomplexan T6PS-TPase proteins are typically large, comprised of more than 1400 amino acids. A number of motifs conserved in both T6PS and TPase domains could be identified from the N- and C-termini of apicomplexan T6PS-TPases (**Figure 3**), which further support their identities.

As indicated by the phylogenetic data below, apicomplexan T6PS-TPases are more closely related to the plant Class II TPS enzymes for which the functional roles in plants are still under investigation [14], [37]–[39]. There was a speculation that some class II proteins from *Arabidopsis thaliana* might be simply TPases (Leyman et al. 2001) [40], and it was also reported that Class II *AtTPS* genes were incapable of complementing the T6PS-deficient *Saccharomyces cerevisiae* (Tps1 mutant) [14]. On the other hand, another study demonstrated that at least one Class II gene (i.e., *AtTPS6*) involved in the regulation of cell shape and plant architecture was able to rescue the yeast Tsp1 mutant phenotype, suggesting that "*AtTPS6* gene may be unique among class II *AtTPS* genes in affecting the cell shape of leaf pavement cells" [37]. Our comprehensive genomic analysis has revealed that apicomplexans, dinoflagellates and ciliates lack discrete T6PS and TPase genes, thus they may only rely on the Class II-like genes to make the trehalose that they possess (as described below).

Most organisms including animals, plants, fungi and bacteria can reutilize trehalose by converting it back to glucose by trehalase [41]–[47]. Among the aveolates, trehalase is present in at least some dinoflagellates and ciliates, but absent in the majority of apicomplexans with a possible exception of *Eimeria* (**Table 1**). Within the partially sequenced *E. tenella* genome, we were able to identify a contig that encodes fragmented protein sequences with significant homology to invertebrate trehalase (i.e., dev_EIMER_contig_00020795), for which its authenticity remains to be experimentally determined. The lack of a trehalase is indicative that the majority of apicomplexans may not recycle trehalose back into its energy metabolism, or they may simply reuse the bifunctional T6PS-TPases in the reverse direction when needed.

### Plant-affinity of apicomplexan trehalose synthetic genes

**Figure 4:**
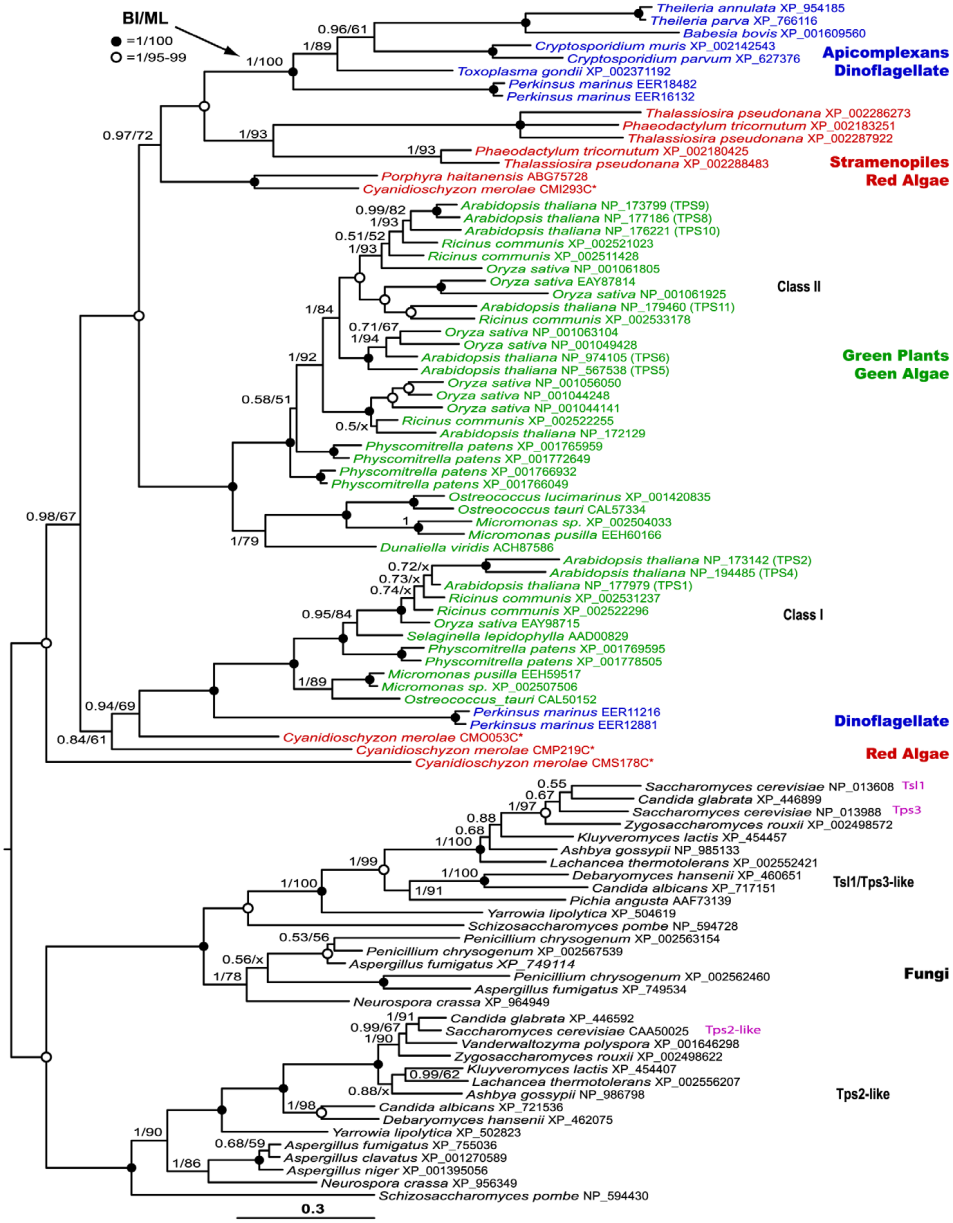
Phylogenetic tree inferred from 93 Class II trehalose-6P synthase-trehalose phosphatase (T6PS-TPase) protein sequences by Bayesian inference (BI) and maximum likelihood (ML) methods. The BI tree is shown here and arbitrarily rooted using fungal sequences as an outgroup. Numbers at nodes are posterior probability (PP) and bootstrap (BP) supporting values determined by BI and ML analyses, respectively. Solid circles indicate nodes with 100% supports by both PP and BP values, while open circles indicates nodes with 100% PP and 95%-99% BP supports.

The availability of trehalose synthetic gene sequences in a large number of diverse taxonomic groups permits a good and unbiased sampling of taxa based on sequence similarity for phylogenetic reconstructions. In our initial **BI** trees inferred from a large dataset, T6PS-TPases were mainly clustered by major taxonomic groups, in which apicomplexans were clustered together with dinoflagellates, red algae, diatoms and then Class II enzymes from green plants and algae (**Figure S1**). The “plant-affinity” of apicomplexan enzymes was further confirmed by **BI** and **ML** analyses of a second dataset with fewer taxa, but a greater number of alignable positions after excluding the more distant prokaryotic and invertebrate sequences (**Figure 4**). Trees inferred from both datasets were robust as the majority of nodes were strongly supported by the PP and BP values in BI and ML analyses. As shown in the second tree (**Figure 4**), the fungal clade (outgroup) is split into two subgroups: one contained regulatory Tsl1/Tps3 subunits, while the other contained Tps2 subunits of the trehalose synthesis complex. Plants, green and red algae, diatoms and dinoflagellates generally possess both Class I and Class II proteins, thus forming distinguished clades. Apicomplexan enzymes joined with *P. marinus* (dinoflagellate), diatoms (stramenopiles) and then red algae to form a monophyletic group as a sister to the Class II enzymes from green plants and algae.

These observations indicate that all Class II enzymes likely originated from a common ancestor, and those in alveolates including apicomplexans are more closely related to the stramenopiles (heterokonts) and red algae than to the green algae and plants. Additionally, the presence of multiple Class II isoforms in many organisms that were placed together within single species, or intermixed with taxonomically closely related species (as exemplified in *A. thaliana* and *S. cerevisiae*) suggests that the trehalose synthetic gene family has undergone expansion by a number of gene duplication events at different taxonomic levels. However, such a gene expansion did not (or at least less commonly) occur in the apicomplexans, as in many cases, only single-copy genes are present in this lineage (**Figure 4**).

**Figure 5:**
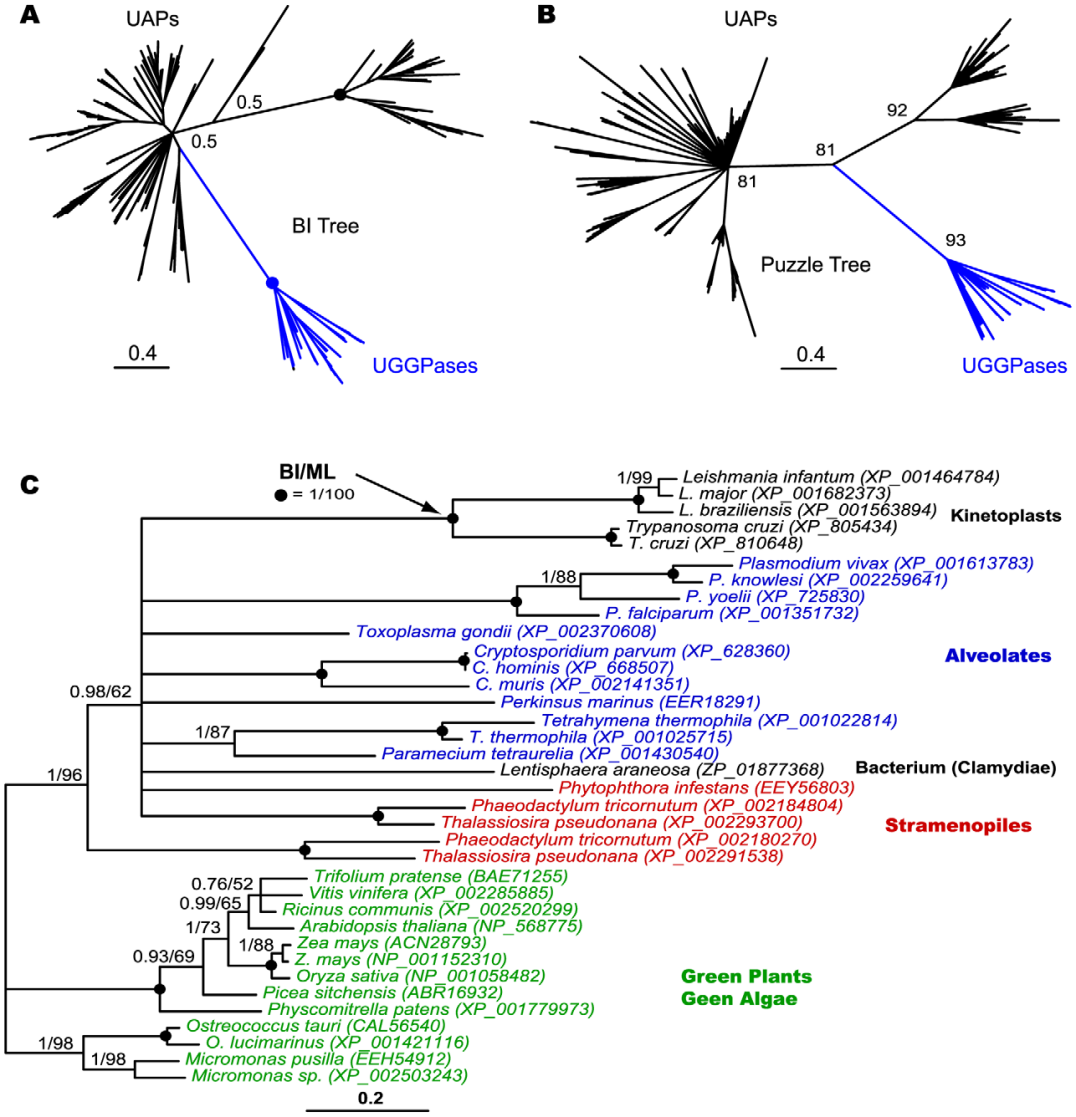
Phylogenetic relationship of apicomplexan UDP-glucose/galatose pyrophosphorylase (UGGPase) among glucosyltransferase family A (GTA) proteins including UDP-*N*-acetylglucosamine pyrophosphorylases (UAPs). **A**) Tree derived from 224 GTA taxa by Bayesian inference (BI) with posterior probability values indicated at major nodes. **B**) Maximum likelihood (ML) tree derived from 224 GTA taxa by quartet puzzling method with quartet puzzling support values indicated at major nodes. **C**) Trees inferred from 93 taxa containing only UGGPase sequences by BI and ML methods using MrBayes and TreeFinder programs, respectively. Numbers at nodes are posterior probability (PP) and bootstrap (BP) supporting values determined by BI and ML analyses. Solid circles indicate nodes with 100% supports by both PP and BP values.

UGGPases are a newly identified group of enzymes capable of making UDP-glucose and UDP-galactose [29], in which UDP-glucose is a precursor for making trehalose. As two closely related members of GTA family proteins, UGGPases share a number of sequence features with UAP enzymes. UGGPases appear to be mainly present in plants, stramenopiles, kinetoplasts and alveolates, whereas UAPs are present in all major prokaryotic and eukaryotic taxonomic groups. Phylogenetic analysis using a large dataset of more than 224 sequences containing both UGGPases and UAPs was performed using BI method, but the runs were not well converged even after more than 10^6^ generations. However, although the poorly converged BI tree was mostly polytomic and unable to resolve the phylogenetic relationships among the majority of sequences, it confidently grouped all UGGPases into a single clade (**Figure 5A**). Additionally, we have also employed a less time-consuming quartet-puzzling analysis using TreePuzzle v5.2 program (*WAG* + *F*_inv_ + *Γ*_(8)_). The puzzle tree produced after 10,000 puzzling steps was better resolved than the BI tree, and again supported the monophyly of UGGPases (**Figure 5B**).

We have also reconstructed trees only from the UGGPase sequences, in which green plants and algae are grouped together, followed by two stramenopiles (**Figure 5C**). However, the remaining sequences formed a polytomic node, in which apicomplexans were only resolved at genus levels. Although sequences within ciliates, stramenopiles and kinetoplastids were better resolved, relationships between these major taxonomic groups were unresolved at phyla levels. These observations indicates that either these sequences do not contain sufficient informative positions for phylogeny and/or there is an insufficient number of available sequences to fully resolve the phylogenetic relationship among UGGPases. It is noticed that plant-type UGGPase genes are also present in kinetoplastids and the chlamydia group bacteria (**Figure 5C**). However, it is not unusual since the “plant” relationship in some enzymes and metabolic pathways in kinetoplastids and chlamydia are well documented [48]–[52]. Collectively, both sequence similarity and phylogenetic data clearly support the notion that apicomplexan UGGPases are of plant affinity and are highly divergent from the closest enzymes in their host animals and humans.

The plant affinity of apicomplexan trehalose synthetic enzymes have previously been implied by the primary genome sequencing projects mainly based on the top hits in BLAST searches [20], [24], and more recently by a large scale metaTIGER phylogenetic tree database project mainly based on a high-throughput PhyML analysis [53], [54]. Our findings are congruent with these earlier observations, but much more robust by employing detailed and comprehensive phylogenetic models with BP/PP supporting values and conserved domain analysis.

### T6PS-TPase gene expression in *Cryptosporidium* is elevated in the late intracellular developmental stages, corresponding to the development of environmental oocysts

**Figure 6:**
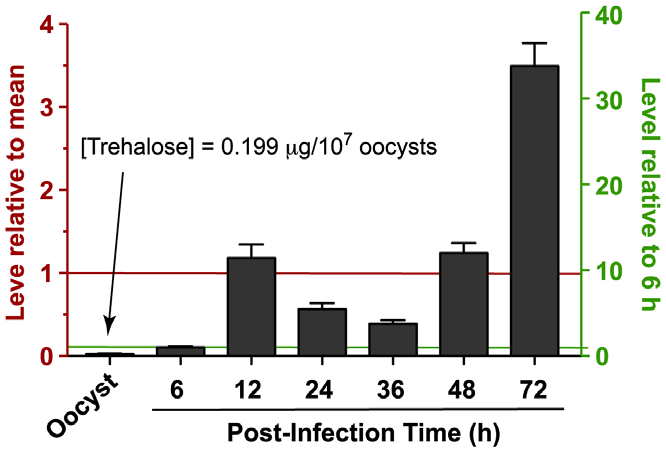
Relative levels of *Cryptosporidium parvum* trehalose-6P synthase-trehalose phosphatase (*CpT6PS-TPase*) gene expressed as determined by real-time quantitative RT-PCR in mature oocysts and intracellular developmental stages in HCT-8 host cells for various post-infection times. The levels of *CpT6PS-TPase* transcripts were first normalized with those parasite 18S rRNA and then displayed as relative to the overall mean (left-side Y-axis) and to the level at 6 h post-infection (right-side Y-axis). The detected trehalose concentration in the parasite oocysts is also indicated.

We hypothesize that trehalose in apicomplexans is an important stress protectant, such as conferring resistance to desiccation or freezing, and secondly, that trehalose functions in the external oocyst stage that is subject to stresses of environmental exposure. Our real-time qRT-PCR analysis with parasite 18S rRNA levels as internal controls indicates that *CpT6PS-TPase* gene is detectable in all life cycle stages, but highly elevated in the late developmental stage. Specifically, at 72 hr post-infection, the relative level of *CpT6PS-TPase* transcripts was ∼3.5-fold higher than the overall mean level, or ∼34-fold higher than the level at 6 h post-infection) (**Figure 6**). The 72 hr post-infection time is correlated with the sexual development and the production of oocysts in *C. parvum*, indicating that this parasite is likely producing a significantly higher level of trehalose for oocysts that would be eventually released into external environment. Similar expression patterns were also observed for genes encoding *Cryptosporidium* oocyst wall proteins (COWPs) [55], [56], and more importantly, also for genes encoding anti-stress related mannitol cycle enzymes that were also found highly expressed in the oocyst production stage in *Eimeria* [57]. One small surprise is the low level expression of *CpT6PS-TPase* gene in the oocysts (**Figure 6**). However, this is not unexpected since trehalose, as a protectant, likely needs to be already synthesized before oocysts are mature and released into natural environment, similar to the oocyst wall proteins.

### Trehalose is present in the *C. parvum* oocysts

Using a trahalase/hexokinase/gluconate-6-phosphate dehydrogenase-coupled spectrophotometric assay, we have determined that trehalose is present in the *C. parvum* oocysts with a concentration at 0.199±0.014 µg/10^7^ oocysts. This roughly equals to 3.5×10^7^ trehalose molecules per oocyst. While we are still refining the assay to detect the trehalose contents in the parasite intracellular life cycle stages, which is complicated by the presence of host cells, the presence of trehalose in the oocysts ultimately confirms that the trehalose synthetic pathway is active in the apicomplexan *C. parvum*. As mentioned earlier, the expression of *CpT6P-TPase* gene was the lowest in the free oocyst stage, but elevated in the later intracellular developmental stages, which implies that trehalose might mainly be synthesized during the formation of oocysts before they were defecated into the external environment.

In summary, we have performed a thorough genomic analysis and reconstructed the trehalose synthetic pathway in the apicomplexans. This pathway is active in *C. parvum* as a gene encoding the key enzyme T6PS-TPase is expressed during the parasite life cycle and trehalose can be detected in the parasite oocysts. Our sequence analysis and phylogenetic recontructions indicate that the two genes involved in the apicomplexan trehalose synthesis (i.e., UGGPase and T6PS-TPase) are plant-type genes. The closest orthologs of these enzymes are those from other alveolates and stramenopiles, suggesting the these genes were probably acquired from the plant-lineage by an ancestral alveolate, retained in some apicomplexan lineages. On the other hand, humans and other mammals lack the Class II bifunctional T6PS-TPase and UGGPase, suggesting that, similar to the enzymes involved in the mannitol cycle in *Eimeria*, these two enzymes (particularly the plant-type T6PS-TPase) may be explored as novel drug targets in *Cryptosporidium* or other apicomplexans. Finally, although in silico analysis and phylogenetic reconstructions can provide some important clues on the functions and evolutionary affinity of proteins under investigation, the true functions and biological roles of enzymes in the apicomplexan trehalose synthetic pathway can only be derived from further biochemical and biological studies.

## Materials and Methods

### Reconstruction of the trehalose synthetic pathway from the genomes

All publicly available apicomplexan genome sequences (completely or nearly completely sequenced) were searched for genes encoding enzymes involved in or connected to the trehalose metabolism. These include 15 species from the genera *Cryptosporidium*, *Toxoplasma*, *Eimeria*, *Plasmodium*, *Theileria* and *Babesia*. Most sequences genome sequences are available at the National Center for Biotechnology Information (NCBI) (http://www.ncbi.nlm.nih.gov/), except for the *Eimeria tenella* genome that is available at the Wellcome Trust Sanger Institute (http://www.sanger.ac.uk/Projects/E_tenella/). Databases at the EuPathDB (http://eupathdb.org/) containing raw and annotated genomes of most apicomplexans (except for those of *Eimeria*, *Theileria* and *Babesia* species) were also searched to ensure quality [58]. In addition to the apicomplexans, searches were also extended to include dinoflagellates and ciliates to gain a more complete picture of the pathway among alveolates.

Targeted enzymes include T6PS, TPase, trehalase, UDP-glucose pyrophosphorylase (**UGPase**) and UDP-galactose/glucose pyrophosphorylase (**UGGPase**). For comparison, enzymes associated with mannitol cycle (i.e., mannitol 1-phosphate dehydrogenase [M1PDH], mannitol-1-phosphatase [M1Pase] and mannitol dehydrogenase [MannDH]) were also searched.

Annotated protein sequences of these enzymes from apicomplexans and other major taxonomic groups (i.e., plants, bacteria, fungi and animals) were used as queries to repeatedly search the nucleotide or translated protein sequences in these databases using TBLASTN and BLASTP algorithms [59], respectively. Hits were then used as queries to search the non-redundant protein sequences and conserved domain databases at NCBI to validate their identities and conserved motifs. Additionally, the mappings and annotation of corresponding apicomplexan genes at the Kyoto Encyclopedia of Genes and Genomes (KEGG) (http://www.genome.jp/kegg/) were also inspected for annotated and missing enzymes [60]. Annotated apicomplexan sequences within the trehalose metabolic pathway at KEGG were retrieved and analyzed for their identities.

Retrieved sequences were aligned using MUSCLE program (version 3.6) [61], [62], and conserved domains and motifs were identified and visualized as sequence logos by bits with the height of symbols within the stack indicates the relative frequency of each amino of the positions using WebLogo 3 (http://weblogo.threeplusone.com/) [63].

### Phylogenetic reconstructions

Phylogenetic reconstructions were performed for the three key enzymes - the bifunctional T6PS-TPase and UGGPase to elucidate their evolutionary histories. To build datasets for phylogenetic analysis, various apicomplexan protein sequences were used as queries to search orthologs from the NBCI non-redundant protein databases. Orthologs were also searched and retrieved from a red alga *Cyanidioschyzon merolae* Genome Project (http://merolae.biol.s.u-tokyo.ac.jp/) [64], [65]. In our initial analysis, nearly all retrieved sequences, up to a few hundreds taxa, were aligned using a MUSCL program (version 3.6). After visual inspection of the alignments with a MacVector program (version 11.0.4), short and nearly identical sequences were removed from the datasets. Neighbor-joining (NJ) trees were first constructed with MacVector program from positions containing no gaps with Poisson-corrected protein distances. These NJ trees were used to guide unbiased selections of sequences that represented all major taxonomic groups (e.g., prokaryotes, fungi, plants, animals, stramenopiles and aveolates) that possessed corresponding enzyme orthologs. In the final datasets, ambiguous and gap-containing positions were excluded from subsequent phylogenetic reconstructions by Bayesian inference (**BI**) and maximum likelihood (**ML**) methods.

The final T6PS-TPase dataset contained 93 taxa and 447 aa positions derived from both T6PS and TPase domains. For UGGPase, we first retrieved orthologs within the GTA superfamily including UAP sequences from the public databases, and built a dataset to contain 224 taxa and 200 amino acid (aa) positions. We also built a smaller dataset containing only UGGPase orthologs with 33 taxa and 228 aa positions. Two datasets were similarly built for T6PS-TPase protein sequences, in which the large dataset contained 140 taxa with 354 aa sampled from all major taxonomic groups. After phylogenetic analysis with BI method, a second dataset was built to include only groups that were more closely related to the apicomplexans (93 taxa), which gave us a larger number of alignable positions (i.e., 472 aa).

Phylogenetic trees were then inferred from the UGGPase and T6PS-TPase datasets by BI and ML methods (and a quartet-puzzling ML method was also applied to the large UGGPase dataset). BI analysis was performed with a parallel version of MrBayes program (version 3.1.2; http://mrbayes.csit.fsu.edu/) [66], in which at least 10^6^ generation of searches were performed with two independent runs, each containing 4 chains running simultaneously. The current trees were saved every 1,000 generations, and the posterior probability (**PP**) values were calculated from the saved BI trees obtained after the runs converged (typically after the first 25% trees were discarded). ML bootstrapping analysis was conducted from 100 replicated sequences with a TreeFinder program (version October 2008; http://www.treefinder.de/) [67], in which consensus ML trees and bootstrap proportion (**BP**) supporting values were calculated from the 100 ML trees by a majority ruling law. Both BI and ML analyses used a WAG amino acid substitution model. Among-site rate heterogeneity considered the fraction of invariance (*F*_inv_) and a discrete 8-rate gamma distribution (i.e., *WAG* + *F*_inv_ + *Γ*_(8)_). Trees were visualized using a FigTree program (version 1.2.3 or 1.3.1; http://tree.bio.ed.ac.uk/software/figtree/) and annotated with an Adobe Illustrator program (version CS4; http://www.adobe.com/).

### Expression profile of T6PS-TP gene in *C. parvum*

To verify that trehalose synthesis is associated with stresses in the apicomplexans, we analyzed the expression pattern of T6PS-TP gene from *C. parvum (CpT6PS-TPase)* by a SYBR-green-based one-step real-time quantitative RT-PCR (qRT-PCR) method as previously described (e.g., [68]–[70]). Total RNA was isolated from various life cycle stages of *C. parvum* (IOWA-1 strain) using an RNeasy isolation kit (Qiagen). These include the mature oocysts (an environmental stage), intracellular developmental stages obtained by infecting human HCT-8 cells for various times (i.e., from 6 to 72 hr post-infection) that cover the parasites development from first and second generations of merogony, to gametogenesis and oocyst production.

The expression levels of *CpT6PS-TPase* gene were determined with an iCycler iQ Real-Time PCR Detection System (Bio-Rad) using primer pairs specific to *CpT6PS-TPase* (i.e., CpT6P-3473, 5′-AGG CAA GCT TTG ACT TGG ATT-3′ and CpT6P-3587R, 5′ TGC TTT TGC TTC TGT TGG AGT-3′) and *C. parvum* 18S rRNA (Cp18S-1011F, 5′-TTG TTC CTT ACT CCT TCA GCA C-3′ and Cp18S-1185R, 5′-TCC TTC CTA TGT CTG GAC CTG-3′). Reactions containing 20 ng of total RNA and 0.2 µM of specified primers were first incubated at 48°C for 30 min to synthesize cDNA, heated at 95°C for 15 min to inactivate reverse transcriptase, and then subjected to 40 thermal cycles (95°C 20 sec, 50°C 30 sec and 72°C 30 sec) of PCR amplification. Because RNA isolated from intracellular parasites contained a large portion of host cell RNA, the levels of parasite 18S rRNA were first used as baselines to normalize those of *CpT6PS-TPase* transcripts in all samples by calculating the ΔC_T_ (i.e., C_T[CpT6P-TPase]_ - C_T[Cp18S]_) for calculating the relative levels of *CpT6PS-TPase* transcripts with 2^−ΔCT^. The relative level of *CpT6PS-TPase* transcripts in each developmental stage was plotted in relative to the overall mean level of all samples (i.e., ratio between individual normalized level and the overall mean) and to the level of the earliest intracellular sample (i.e., 6 h post-infection).

### Detection of trehalose in *C. parvum*

Fresh *C. parvum* oocysts (IOWA-1 strain) were purchased from Bunch Grass Farm (Deary, Idaho, USA) and stored at 4°C until use. Before the assay, oocysts were treated with 10% Clorox in ice for 10 min, washed for 5–8 times with water by centrifugation, and purified by a Percoll gradient centrifugation protocol as described elsewhere. To detect trehalose, 2×10^8^ oocysts were suspended in 250 µl PBS (pH 7.2) and subject to 5 times of freeze/thaw cycles. The disrupted oocysts were treated at 65°C for 20 min to inactivate enzymes, and then centrifuged at 10,000×g to remove insoluble materials. A spectrophotometry-based trehalose detection kit purchased from Megazyme International Ireland Limited (catalog # K-TREH) was used in this study. The kit detected trehalose by a three-enzyme coupled assay, in which trehalose was first hydrolyzed to D-glucose that was then phosphorylated to glucose-6-phosphate (G-6-P). Finally, G-6-P was oxidized to gluconate-6-phosphate by G-6-P dehydrogenase (G6PDH) using NADP^+^ as an electron receiver, in which the formation of NADPH could be monitored by measuring the increase of absorbance at 340 nm (OD_340_).

The assay was performed according to the manufacturer's protocol, but the reaction volumes were reduced to 63 µl using a 384-well UV transmissible microplate to save parasite materials. The OD_340_ values were measured with a Multiskan Spectrum Microplate Spectrophotometer (Thermo Scientific). For each sample, the difference in the OD_340_ values between reactions with and without trehalase were used to calculate trehalose concentration against a standard curve generated with serial concentrations of trehalose under the same experimental conditions. In this assay, we have noticed that heat-inactivation of oocyst extracts was critical as active enzymes in the non-inactivated samples could rapidly consume NADPH to the background level.

## Supporting Information

Figure S1 Unrooted tree inferred from T6PS-TPase protein sequences (93 taxa, 444 amino acid positions) by Bayesian inference (BI) method using the same amino acid substitution model and the consideration of rate heterogeneity as described in the Methods section. Solid circles indicate select major nodes that were 100% supported by posterior probability (PP) values. Only representative genus names are labeled to indicate taxonomic affiliations of major clusters.
(0.39 MB JPG)
