# Supplementary figures and images for: Plant-Type Trehalose Synthetic Pathway in Cryptosporidium and Some Other Apicomplexans
Source: PLoS One. 2010 Sep 7;5(9):e12593. doi: 10.1371/journal.pone.0012593 (PMC2935371; doi:10.1371/journal.pone.0012593)

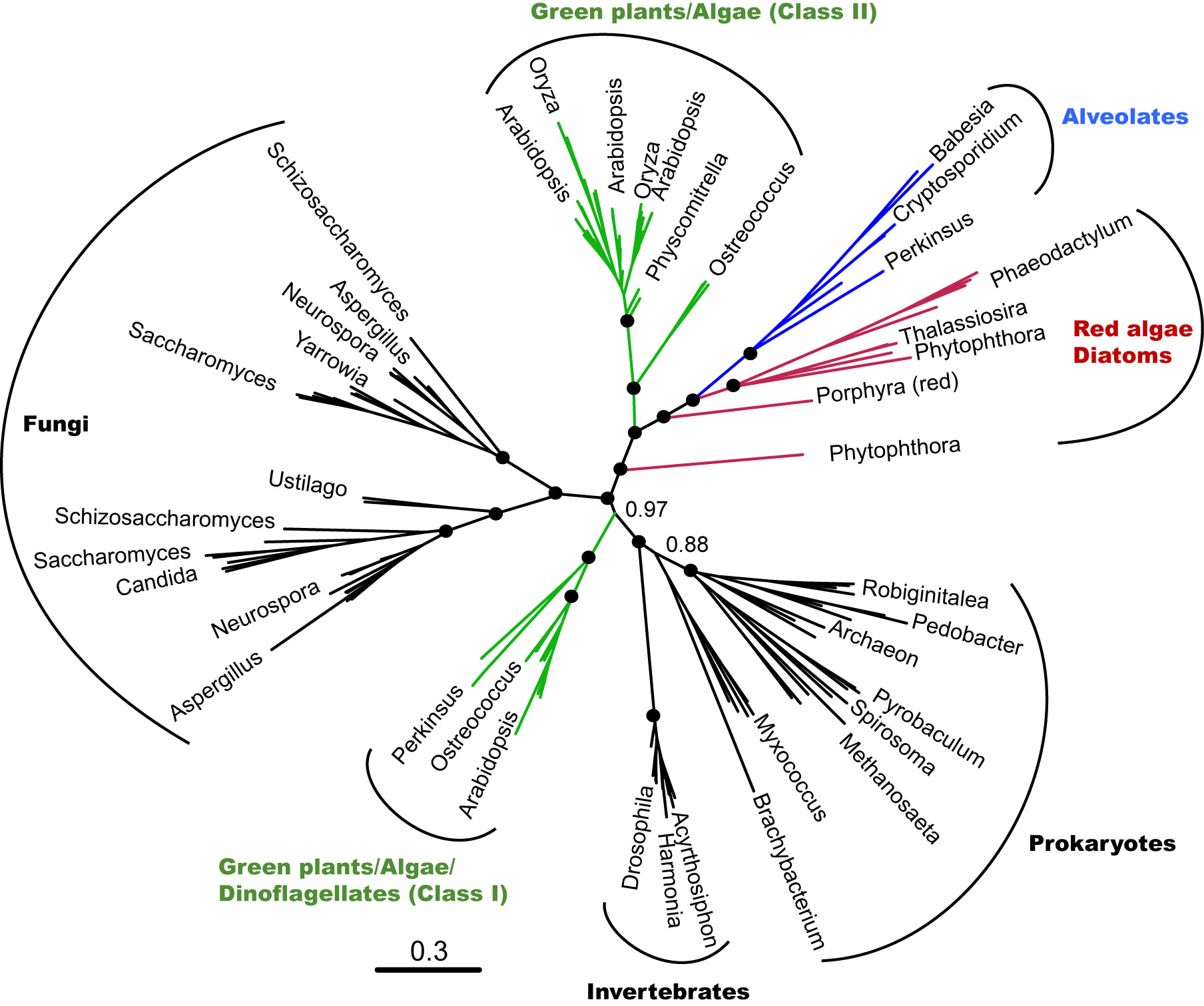

Supplement: Figure S1 — Unrooted tree inferred from T6PS-TPase protein sequences (93 taxa, 444 amino acid positions) by Bayesian inference (BI) method using the same amino acid substitution model and the consideration of rate heterogeneity as described in the Methods section. Solid circles indicate select major nodes that were 100% supported by posterior probability (PP) values. Only representative genus names are labeled to indicate taxonomic affiliations of major clusters. (0.39 MB JPG) [file pone.0012593.s001.jpg]
